# Novel Effects of Leonardite-Based Applications on Sugar Beet

**DOI:** 10.3389/fpls.2021.646025

**Published:** 2021-03-18

**Authors:** Maria C. Della Lucia, Giovanni Bertoldo, Chiara Broccanello, Laura Maretto, Samathmika Ravi, Francesco Marinello, Luigi Sartori, Giovanni Marsilio, Andrea Baglieri, Alessandro Romano, Mauro Colombo, Francesco Magro, Giovanni Campagna, Giuseppe Concheri, Andrea Squartini, Piergiorgio Stevanato

**Affiliations:** ^1^Department of Agronomy, Food, Natural Resources, Animals and Environment, University of Padova, Padua, Italy; ^2^Department of Landscape and Agro-Forestry Systems, Agripolis, University of Padova, Padua, Italy; ^3^Department of Agriculture, Food and Environment, University of Catania, Catania, Italy; ^4^Plant Protection and Certification Centre, Council for Agricultural Research and Economics, Lonigo, Italy; ^5^Research Institute for Industrial Crops, Council for Agricultural Research and Agricultural Economics, Rovigo, Italy; ^6^Sipcam Oxon S.p.A., Milan, Italy; ^7^Cooperativa Produttori Bieticoli (COPROB), Bologna, Italy

**Keywords:** sugar beet, leonardite, 16S rRNA metabarcoding, gene expression, sugar yield

## Abstract

The present study aimed to explore the effects of foliar application of a leonardite-based product on sugar beet (*Beta vulgaris* L.) plants grown in the field. The approach concerned the evaluation of the community compositional structure of plant endophytic bacteria through a metabarcoding approach, the expression level of a gene panel related to hormonal metabolism and signaling, and the main sugar beet productivity traits. Results indicated that plants treated with leonardite (dosage of 2,000 ml ha^–1^, dilution 1:125, 4 mg C l^–1^) compared with untreated ones had a significant increase (*p* < 0.05) in (i) the abundance of *Oxalicibacterium* spp., recognized to be an endophyte bacterial genus with plant growth-promoting activity; (ii) the expression level of *LAX2* gene, coding for auxin transport proteins; and (iii) sugar yield. This study represents a step forward to advance our understanding of the changes induced by leonardite-based biostimulant in sugar beet.

## Introduction

Biostimulant products, applied to soil or plants, are recognized for improving plant health, quality, and yield (Nardi et al., 2018). They have been shown to influence plant metabolism through the enhancement of photosynthesis, water use efficiency, nutrient uptake, and assimilation (Calvo et al., 2014; Yakhin et al., 2017). Although the study of biostimulation mechanisms is still an ongoing task, available research highlighted a hormone-like activity and an enhancement of root and organ growth and development (Canellas et al., 2011). Moreover, biostimulants have an important role in promoting tolerance to abiotic stresses and plant recovery (Halpern et al., 2015; Van Oosten et al., 2017). Humic substances (HSs), such as leonardite, have prominent importance among biostimulant products. They are a dark brown natural organic compounds, ubiquitous in water, soil, and sediments (Piccolo, 2002). Particularly, leonardite, originating from the atmospheric oxidation of lignite, is very rich in humic acids (David et al., 2014). Leonardite application has been shown to improve nutrient uptake, such as Fe, N, and K, and increase plant yield and quality (Ece et al., 2007; Fascella et al., 2015; Cieschi et al., 2017). Therefore, leonardite is generally used in agriculture as a soil conditioner, increasing the permeability of the stem cell membrane, nutrition rate, fruit quality, and crop yield (Ratanaprommanee et al., 2017). An improved production has been reported for leonardite-treated cherry, potato, corn, and ornamentals (Eyheraguibel et al., 2008; Sanli et al., 2013; Fascella et al., 2018; Demirer, 2019). Sugar beet (*Beta vulgaris* L.) plays a key role in the agricultural and economic scenario of 52 countries. In 2017, the world area harvested with sugar beet reached almost 5 Mh for a total production of 314 Mt (Food and Agriculture Organization (FAO), 2019), and the increasing trend is to move toward a sustainable cultivation. In this context, biostimulant products are classified as ecofriendly, minimizing the agricultural impact on the environment. Furthermore, these products not only protect microbes already present in the soil but also foster the growth of new rhizosphere bacteria communities and the related soil enzymatic activity (Du Jardin, 2015). Thus, the use of biostimulants is based on the knowledge of plant root and shoot bacterial communities.

The compositional structure of plant endophytic microbes is influenced by many factors. External environmental conditions, climate, biotic stresses, human practices, and the soil environment are the most important key factors altering the composition of plant endophytic communities (Reinhold-Hurek et al., 2015). The role of endophytic bacteria is crucial. Several studies revealed protective function from plant abiotic stresses, accelerating plant immune response following pathogen infection (Miliute et al., 2015). Furthermore, they can promote plant growth, development, and nutrient uptake (Liu et al., 2017). However, significant knowledge gaps remain, involving the cross-talk between plant and microbes and how the microbiome modulates gene expression in the plant (Liu et al., 2020).

Analysis of plant microbial communities requires suitable techniques and reproducible protocols. A rapidly emerging technique to explore complex bacterial populations is presented by the 16S rRNA gene metabarcoding. This approach, common between different sequencing platforms, involves the PCR amplification of the most taxonomically informative region of 16S rRNA gene followed by high-throughput sequencing. The 16S gene includes nine hypervariable regions (V1—V9) that are taxon-specific, flanked by conserved sequences. The selection of the most informative region is still a matter of scientific debate. V3 and V4 are the most commonly used regions for taxon identification (Yarza et al., 2014).

The present work aimed to explore the effects of leonardite treatment on sugar beet. For this purpose, we firstly compared the microbiome profiles of plants cultivated in hydroponics and field conditions. Then, we exploited the effect of foliar application on plants grown in the open field. Therefore, we investigated (i) the consequences of leonardite application on the composition of plant endophytic communities, (ii) the expression level of key genes related to hormonal and signaling metabolism, (iii) and its impact on yield traits using sugar beet (*B. vulgaris* L.) as a model crop.

## Materials and Methods

### Plant Material

The sugar beet variety used for the experimental trials, both in the field and in hydroponics, was Smart-Briga (KWS, Einbeck, Germany), diploid and resistant to the herbicide Conviso, Cercospora leaf spot, Rhizomania, and nematodes.

### Field Experiment

The field trials were carried out in four locations for 6 months, between March and August 2020. The geographical coordinates of the four locations involved are Pozzonovo, Padua, Italy (45°10’49.7”N, 11°47’48.0”E); Loreo, Rovigo, Italy (45°04’33.6”N, 12°10’36.2”E); Cavarzere, Venezia, Italy (45°06’37.7”N, 12°03’05.1”E); and San Martino di Venezze, Rovigo, Italy (45°06’12.9”N, 11°53’52.5”E). An experimental design constituted of four randomized blocks was applied. Each of the randomized blocks was divided into four subplots whose size was 2.7 × 10 m. A control plot was placed outside the randomized block, and plants were kept without treatments. Plants were subjected to foliar spray treatments with leonardite solution using a dosage of 2,000 ml ha^–1^ (dilution 1:125, 4 mg C l^–1^). The novel leonardite formulation and non-commercial product used in this work was provided by Sipcam SpA (Italy). The leonardite formulation was analyzed by combustion (Elementar vario MACRO CNS, Elementar Analysensesystemse GmbH, Germany) for C, N, and S contents, ionomic analysis (inductively coupled plasma optical emission spectrometry, SPECTRO ARCOS II MV, SPECTRO, Germany) for elemental analysis, and NMR analysis (solid-state 13C MAS NMR spectra, fully proton-decoupled using a Bruker Avance II 400 MHz instrument, Bruker Corp., United States) for spectra and the distribution of the diverse forms of carbon. The results of this analysis were previously described by Barone et al. (2019). The first application was set for the stage BBCH 38 (leaves cover 80% of the ground), the second treatment was performed 40 days after the first, and the last treatment was applied 20 days after the second one. The untreated control plants were sprayed only with water. A 50-l backpack sprayer was used to uniformly distribute the leonardite solution. Four biological replicates consisting of three-leaf discs taken by plants randomly picked, inside the same subplot, were collected 48 h after treatment. Samples of approximately 50 mg of leaf tissue were placed in dry ice and taken to the laboratory for DNA extraction.

### Hydroponic Experiment

Sugar beet seeds were sterilized by dipping in 76% ethanol for 5 min. The washing procedure with distilled water was repeated three times. To promote germination, seeds were kept inside a growing chamber in the dark on distilled water-moistened filter paper for 48 h at 25°C. Six days after germination, plants were transferred inside 500 ml glass pots with complete Hoagland solution (Arnon and Hoagland, 1940). After 6 days, plants were divided into two different pots containing, respectively, 1 ml/l of leonardite (treated plants) and complete Hoagland solution (control plants). Leaf sampling was done 2 days after leonardite treatment. The experiment was repeated three times for validation aims.

### DNA Extraction

DNA was extracted from 50 mg of fresh leaf material. Samples were homogenized inside the collection microtubes with 300 μl of Buffer RLT and 3 mm stainless steel beads. The homogenization step involved the use of Tissue Lyser (Qiagen, Hilden) for 5 min at 30 Hz. Homogenized samples were then transferred in a 96-well S-block plate containing also 200 μl of isopropanol and 20 μl of MagAttract magnetic beads (Qiagen). This plate was used for automatic DNA extraction using Biosprint 96 (Qiagen) together with five other plates respectively composed of 500 μl of Buffer RPW, 500 μl of 0.02% Tween, and two plates filled with 500 μl of 96% ethanol. DNA was eluted in 100 μl of nuclease-free water. Nucleic acid quantification was performed using Qubit (Thermo Fisher Scientific, Carlsbad, CA) with Qubit DNA High Sensitivity Assay Kit (Thermo Fisher Scientific).

### RNA Extraction

mRNA was isolated using Dynabeads mRNA Direct Micro Kit (Thermo Fisher Scientific) according to the manufacturer’s instructions, starting from 50 mg of leaf material. mRNA was immediately analyzed with qPCRBIO SyGreen 1-step kit (Resnova-PCR Biosystem).

### Metabarcoding of Bacterial 16S rRNA Gene by High-Throughput Sequencing

Library preparation was carried out using the 16S Ion Metagenomics Kit (Thermo Fisher Scientific). Briefly, the protocol consists of a first PCR amplification using two different primer sets (V2, V4, V8 and V3, V6, V7, V9) for the amplification of seven different hypervariable regions. The PCR program consisted of an initial denaturation of 95°C for 10 min, followed by 25 cycles of 95°C for 30 s, 58°C for 30 s, 72°C for 20 s, and a hold stage at 72°C for 7 min. Amplicons were quantified and pooled together to obtain a final concentration of 30 ng μl^–1^. Subsequently, the protocol involved the use of the Ion Xpress Plus Fragment Library Kit (Thermo Fisher Scientific) and Ion Express Barcode Kit (Thermo Fisher Scientific) for bar code ligation. The library was amplified with six cycles of PCR at 58°C for 15 s and 70°C for 1 min, then 4°C for up to 1 h. The library was diluted to a concentration of 25 pM and used to prepare the template positive Ion sphere particles with Ion One Touch 2 instrument (Thermo Fisher Scientific). The enrichment process was done with the Ion ES instrument (Thermo Fisher Scientific) and the sequencing with Ion GeneStudio S5 using the Ion 520 chip kit (Thermo Fisher Scientific). The data were analyzed using the Ion Torrent Suite software, and the taxonomical assignment was performed by comparing operational taxonomic units (OTUs) against the Greengenes database (version 13.5) and the curated MircoSeq reference library v2013.1 on the Ion Reporter cloud (Thermo Fisher Scientific).

### Real-Time PCR for Bacterial Detection

The obtained bacterial sequences were used to design Real-Time PCR primers with the software Primer Express V3.0 (Thermo Fisher Scientific). The primer sequences used in this work are reported in Table 1. Real-Time PCR was conducted using QuantStudio 5 (Life Technologies, United States) with the following mix: 5 μl of SYBR Green Real-Time PCR Master Mix, 0.1 μl of forward primer, 0.1 μl of reverse primer, 1.4 μl of nuclease-free water, and 1 μl of each sample. The PCR program was set as follows: 10 min of preincubation at 95°C and 50 cycles of 15 s at 95°C and 1 min at 60°C.

**TABLE 1:** List of forward and reverse primer sets used for quantification of bacterial genera by Real-Time PCR on leonardite-treated and untreated samples.

Name	Forward primer 5′–3′	Reverse primer 5′–3′
*Pseudomonas*	GCGCGTAGGTGGCTTGATAA	GGATGCAGTTCCCAGGTTGA
*Burkholderia*	CCTCTGCCATACTCTAGCCC	ATGTGAAATCCCCGGGCTTA
*Oxalicibacterium*	GCGCAACCCTTGTCATTAGT	TGTCACCGGCAGTCTCATTA
*Massilia*	CAATGCCGCGTGAGTGAA	GAACCGTTTCTTCCCTGACAAA
*Propionibacterium*	GGGTTAAGTCCCGCAACGA	ACCATAACGTGCTGGCAACA
*Methylobacterium*	CTTCCGGTACCGTCATTATCG	GTGATGAAGGCCTTAGGGTTGT
*Hymenobacter*	AGGTGGCCCCGCAAGT	TCCATGGCAGTTCTGTAGTTGAG
*Xanthomonas*	AAGGTGGGGATGACGTCAAG	TGTGTAGCCCTGGTCGTAAG

### Real-Time Quantitative RT-PCR for Expressed Plant Genes

Eight sugar beet genes were used to test leonardite effects on plants. Primer design with Primer Express V3.0 (Thermo Fisher Scientific) was done starting from mRNA sequences downloaded from RefBeet_1.2^1^. Table 2 shows the complete list of genes, their functional category, and gene product. Quantitative RT Real-Time PCR amplification and detection were conducted on a Quant Studio 12K Flex Real-Time PCR (Thermo Fisher Scientific) using qPCRBIO SyGreen 1-step kit (Resnova-PCR Biosystem). The 10 μl of reaction mixture contained 5 μl of SYBR Green, 0.5 μl retrotranscriptase, 0.4 μl of forward and reverse primers, 0.7 μl of nuclease-free water, and 1 μl of RNA. The threshold cycle (Ct) values obtained were normalized against the average transcript abundance of three housekeeping genes (*Tubulin*, Bv2_037220_rayf; *GAPDH*, Bv5_107870_ygnn; *Histone* H3, Bv6_127000_pera) using the formula: 2^–ΔCt^ in which ΔCt is obtained from the difference between the Ct of the target gene and the Ct of the control gene (Livak and Schmittgen, 2001; Schmittgen and Livak, 2008).

**TABLE 2:** Details of genes used for quantitative RT Real-Time PCR showing their functional category and gene product.

Gene	Category	Gene product
*AREB1*	Hormone metabolism	Abscisic acid-insensitive 5-like protein
*HAB1*	Hormone metabolism	Serine/threonine phosphatases Mg dependent
*AHG3*	Hormone metabolism	Phosphatases 2C
*AUX1*	Hormone metabolism	Auxin transporter-like protein 1
*ATTIR1*	Hormone metabolism	Protein transport inhibitor response 1, auxin binding
*LAX2*	Hormone metabolism	Auxin transporter-like protein 2
*PIN3*	Hormone metabolism	Auxin efflux membrane carrier protein, component 3
*CSD2*	Hormone metabolism	Superoxide dismutase [Cu-Zn]

### Yield Traits

The effect of leonardite on sugar beet yield traits such as root yield, sugar yield, and processing quality-related traits were evaluated between March and August 2020 in Pozzonovo, Padua, Italy (45°10’49.7”N, 11°47’48.0”E). The experimental design was divided into four randomized blocks, each one divided into four subplots whose size was 2.7 × 10 m. Outside the randomized block, a control plot was placed, and plants were kept without any treatments. The foliar spray treatments with leonardite solution were done using a dosage of 2,000 ml ha^–1^ (dilution 1:125, 4 mg C l^–1^). Topped sugar beets from each subplot were collected after BBCH 49 (beet root has reached harvestable size) and analyzed to detect the mean of root yield, sugar yield, and processing quality-related traits as influenced by leonardite application. Roots from each collected plant were washed, and using a special sawing machine (AMA-KWS, AMA Werk GmbH, Alfeld, Germany), 1 kg of micronized tissues (brei) was obtained. About 70 g of representative homogenized brei samples were immediately frozen at −40°C. Sugar content and the main non-sugars were analyzed after cold digestion of the brei in lead acetate 0.75% (w/w) solution (Schneider, 1979) using an automated brei mixer (Venema Automation b.v., Groningen, Netherlands). To quantify the sugar content, a Thorn-Bendix 243 polarimeter (Bendix Corp., Nottingham, United Kingdom) was used, whereas K and Na concentrations were measured by a flame photometer (Model IL 754, Instrumentation Laboratory S.p.A., Milan, Italy). The α-amino N was quantified by colorimetric analysis (PM2K; Carl Zeiss GmbH, Oberkochen, Germany) following the procedure proposed by Kubadinow and Wieninger (1972) and Stevanato et al. (2010). The purity was calculated as the percentage of sugar from the roots extractable by the factory according to Wieninger and Kubadinow (1971) and Stevanato et al. (2010).

### Data Analysis

Data analysis of community compositional structure of plant endophytic bacteria was conducted using Ion Torrent Suite software 5.16. This included the use of BaseCaller module to filter out low-quality sequences marked during the signal processing step followed by base calling, barcode assignment, and adaptor trimming at 3’ end. The preprocessed fastq files were analyzed using Quantitative Insights into Microbial Ecology (QIIME) 1.9.1 pipeline. OTU clustering was done using a unique read abundance threshold of 10 and 97% sequence similarity against the curated Greengenes database v.13.8 and Curated MicroSEQ 16S Reference Library v2013.1. Microbial diversity was assessed using alpha and beta diversity using QIIME. The relative abundance of OTUs was calculated for both the family and genus level. Permutational multivariate analysis of variance (PERMANOVA), to test significance between groups, was performed using QIIME.

Data analysis of expression level of the gene panel and the main sugar beet productivity traits was conducted using Statistica v13.4 (Dell, Round Rock, TX, United States). Significant differences among the mean values were evaluated with Student *t*-test followed by *post hoc* analysis (Duncan’s test). Significance was estimated at the *p* < 0.05 level. Data are expressed as mean ± standard error of the mean.

## Results

Bacterial 16S rRNA metabarcoding was performed on 14 untreated samples. We chose to sequence two groups of untreated plants, seven coming from the field (located in Pozzonovo, Padua, Italy) and seven grown in hydroponic solution, to study and compare the microbiome composition of sugar beet grown in two different environments without any treatment. Sequences have been deposited in the European Nucleotide Archive (ENA) browser under accession numbers PRJEB42500 and ERP126366.

**FIGURE 1:**
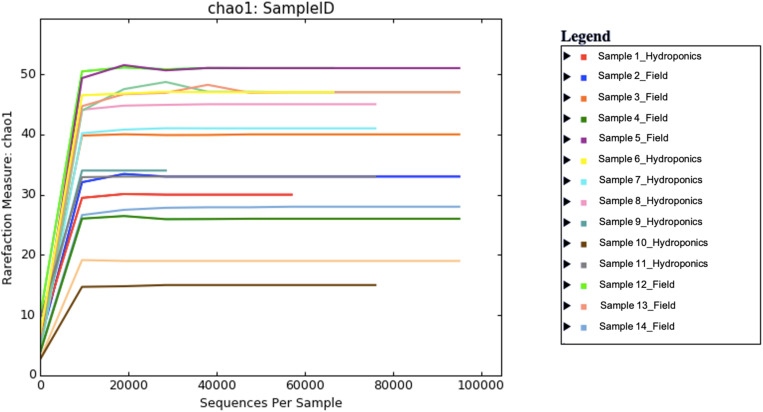
Alpha diversity in seven field and hydroponics-grown plants calculated with the Chao diversity index.

**FIGURE 2:**
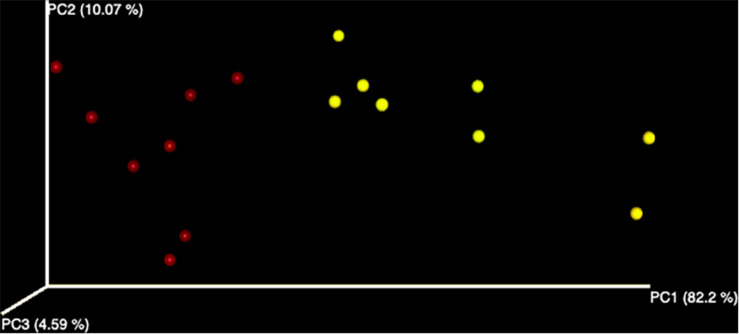
Evaluation of beta diversity in field (red) and hydroponic (yellow) plants. The principal component analysis was performed using Quantitative Insights into Microbial Ecology (QIIME).

A total number of 2,145,785 paired-end sequences were obtained, with an average length of 258 bp, and among them, 635,152 (29.6%) were rejected after the filtering process with the Torrent Suite software. Sequences were clustered into 139 OTUs at 97% identity cutoff. The remaining OTUs, divided into 34 different families and 37 genera, were subjected to the characterization of the endophytic bacterial communities. Alpha diversity, corresponding to the number of species or OTUs within samples (Willis, 2019), showed the highest number of sequences in samples grown in the field compared to hydroponics using the Chao indexes (Figure 1). A principal component analysis based on Euclidean distance was used to show how bacterial communities were distributed between field and hydroponics (Figure 2). Plants grown in hydroponic conditions (yellow dots) clustered separately from plants grown in the field (red dots) (PERMANOVA, *p* < 0.05).

**FIGURE 3:**
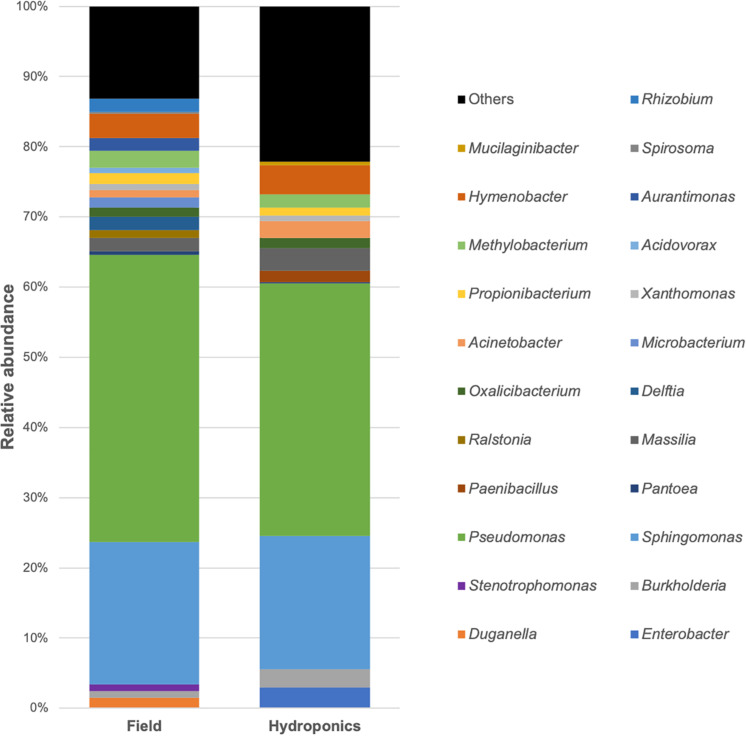
Relative sequence abundance of bacterial genera associated with field and hydroponics-grown plants. The most represented operational taxonomic units (OTUs), with relative abundance higher than 1%, are reported. OTUs with less than 1% are assigned as “Others.”

The complete microbial profiles generated are shown in Figure 3. Bar-plot analysis showed that the majority of OTUs in the two groups were assigned to the genera *Pseudomonas*, followed by *Sphingomonas*, *Hymenobacter*, and *Methylobacterium*, as reported also by the percentage listed in Table 3. The minority of the OTUs found belonged to *Propionibacterium*, *Burkholderia*, *Massilia*, *Oxalicibacter*, and *Xanthomonas* (Table 3). Moreover, the bar plot represented a remarkable variability in the field-grown plants at the genus level. This variability is directly related to a higher number of genera identified, 20 in the field-grown plants compared to the 14 genera identified in hydroponics-grown ones. Particularly, these additional genera included *Duganella*, *Stenotrophomonas*, *Ralstonia*, *Delftia*, *Microbacterium*, *Acidovorax*, *Aurantimonas*, *Spirosoma*, and *Rhizobium.* In Figure 3, “Others” represents bacterial genera that formed less than 1% of the total abundance.

**TABLE 3:** Mean relative abundance (%) in each group at the genus level.

Genera	Field (%)	Hydroponics (%)
*Pseudomonas*	47.0	46.2
*Sphingomonas*	23.6	24.4
*Hymenobacter*	4.0	5.3
*Methylobacterium*	2.9	2.4
*Massilia*	2.2	4.1
*Propionibacterium*	1.8	1.4
*Oxalicibacterium*	1.4	1.9
*Burkholderia*	1.1	3.3
*Xanthomonas*	1.0	1.0

**Bacteria with relative abundance higher than 1.0% are reported.**

Specific Real-Time PCR primer pairs were designed to detect eight genera, constituting the core microbiome of sugar beet, on leaf samples collected under field conditions (in four different locations) on 48 h leonardite-treated plants and untreated ones. All genera tested by Real-Time PCR were detected in both treated and untreated plants, without showing any significant variation, with exception of *Oxalicibacterium* spp. The average threshold cycle obtained for untreated samples was 24.20 with a standard error of 0.33, while samples treated with a dosage of 2,000 ml ha^–1^ (dilution 1:125) showed an average of 23.32 and a standard error of 0.29. Ct resulted from the mean of three biological replicates. Using a *p*-value threshold at 0.05, the treated samples have a significantly lower Ct value (indicating higher amounts of the template related to the presence of *Oxalicibacterium* spp.) compared to the untreated ones.

Quantitative RT Real-Time PCR was carried out to identify changes in gene expression profile between untreated and treated plants of the four locations. The selected genes had been detected in a previously published paper by Barone et al. (2019), where they were found responsive to leonardite treatment in hydroponic conditions. Among the complete dataset of 53 genes, we choose the ones involved in hormone metabolism. Table 4 shows the percentage of variation in the gene expression level of treated samples with respect to the untreated ones. Samples were collected after 24 h from leonardite treatment using a dosage of 2,000 ml ha^–1^ (dilution 1:125). One of the analyzed genes, *LAX2*, showed a significantly different level of expression (*p* < 0.05) in treated vs. untreated samples. This gene encodes for an auxin transport protein. Particularly, 24 h after the leonardite application, an expression level of 38% over the control of the *LAX2* was observed.

**TABLE 4:** Percentage variation in the gene expression level of treated samples with respect to the untreated ones.

Genes	Percentage of variation	*p*-value
*AREB1*	31%	n.s.
*HAB1*	8%	n.s.
*AHG3*	16%	n.s.
*AUX1*	-4%	n.s.
*ATTIR1*	13%	n.s.
*LAX2*	38%	0.025
*PIN3*	-7%	n.s.
*CSD2*	37%	n.s.

**Student t-test was applied to verify the statistical significance between groups (p < 0.05; n.s., not significant). Samples were collected after 24 h from leonardite treatment using a dosage of 2,000 ml ha^–1^ (dilution 1:125), in four different locations.**

Table 5 shows yield values and quality parameters as obtained from laboratory analyses on leonardite-treated and untreated sugar beet coming from Pozzonovo, Padua, Italy. The sugar yield of plants treated with leonardite (12.30 ± 1.13 t ha^−1^) was significantly higher (*p* < 0.05) compared to that of the untreated ones (11.40 ± 1.56 t ha^−1^). No significant differences can be observed in quality parameters of juice such as Na, K, α-amino N content, and sugar purity.

**TABLE 5:** Mean of root yield, sugar yield, and processing quality-related traits in leonardite-treated and untreated sugar beet grown in Pozzonovo, Padua, Italy (45°10’49.7”N, 11°47’48.0”E).

Samples	Root yield	Sugar yield	Potassium	Sodium	α-amino N	Sugar
	(t ha^–1^)	(t ha^–1^)	(meq%°S)	(meq%°S)	(meq%°S)	purity (%)
Untreated	75.70 ± 5.10	11.40 ± 1.56	24.38 ± 1.91	8.07 ± 0.90	6.69 ± 0.78	93.70 ± 8.19
Treated	80.70 ± 7.23	12.30* ± 1.13	23.54 ± 2.54	7.73 ± 0.65	7.04 ± 0.89	93.80 ± 10.17

**These measurements were performed on four replicates with 60 plants each. ANOVA was used to evaluate differences between the two treatments with a 0.05 p-value threshold. Mean values followed by asterisk differ significantly from untreated samples (p < 0.05).**

## Discussion

Maintaining a healthy environment, while increasing plant yield and quality, is one of the key aspects of sustainable agriculture. The application of chemical pesticides and fertilizers can undermine soil quality and invertebrate population (Liu et al., 2015). Therefore, the scientific community is studying the role and specific effects of organic plant biostimulants as a gradual and promising replacement of chemical products.

Among biostimulants, leonardite, due to the high percentage of humic acids, is considered a bioactive compound suitable to preserve soil integrity (Turgay et al., 2010). Organic molecules (phenolic and alcohol compounds) contained in leonardite can be used by microbes as a source of nitrogen and carbon (Conselvan et al., 2017; Zhang et al., 2020). Consequently, the microbiome change following leonardite applications may be useful in elucidating the mechanism of action of this product (Yu et al., 2015). Therefore, the monitoring of bacterial species and their relative abundance is fundamental to understand the changes induced by biostimulant application.

In this study, the 16S rRNA metabarcoding analysis was performed on the pretreated microbiota of seven sugar beets grown in the field and seven grown in hydroponics. This comparison revealed nine shared bacterial genera between the two groups of plants. *Pseudomonas*, *Sphingomonas*, *Methylobacterium*, *Propionibacterium*, *Burkholderia*, *Massilia*, *Oxalicibacterium*, *Hymenobacter*, and *Xanthomonas* constituted the core microbiome of seedlings grown in the two different environments. These, being found also in hydroponically grown seedlings, qualify as plant-borne and seed sterilization-resistant endophytes. As a result, these bacteria outline the seed microbiome of the sugar beet genotype used to compare the changes brought by leonardite treatments. These common bacteria are recognized to be seed endophytes with plant growth-promoting activity (Truyens et al., 2015), such as *Pseudomonas* and *Sphingomonas*, found also to be the most abundant genera. Other genera, including *Propionibacterium* and *Burkholderia*, are involved in seed germination and root and shoot growth (Johnston-Monje and Raizada, 2011; Rodríguez et al., 2020). Among total bacteria found through sequencing, many of them were unique of field-grown sugar beet, originating from soil and environment. These are *Duganella*, *Stenotrophomonas*, *Ralstonia*, *Delftia*, *Microbacterium*, *Acidovorax*, *Aurantimonas*, *Spirosoma*, and *Rhizobium.* They can be mostly divided into disease suppressive, such as *Duganella*, *Microbacterium*, *Rhizobium*, *Delftia*, and *Stenotrophomonas* that also have beneficial activity on plant growth and, on the other hand, *Acidovorax* and *Ralstonia* are recognized to be plant pathogens (Bergna et al., 2018; Woźniak et al., 2019).

The shared bacteria between the two groups were analyzed using quantitative Real-Time PCR on leonardite-treated and untreated sugar beet. Specific primers were designed to quantify their abundance. The results obtained showed that *Oxalicibacterium* spp. revealed a significant increase in abundance in plants treated with leonardite. *Oxalicibacterium* spp. belongs to the *Oxalobacteraceae* family, and among this family, we detected also the genus *Massilia*. *Massilia* is the richest genus of the *Oxalobacteraceae* family, isolated from roots and leaves, with plant growth-promoting activity and disease-suppressive abilities, while *Oxalicibacterium* is considered the most specialized oxalate degrader (Bonanomi et al., 2018; Raths et al., 2020). Oxalate is a secondary metabolite, widely reported in plants and soils, and a major component of root exudate with a key role in the recruitment of soil microbial species (Martin et al., 2012; Baldani et al., 2014). Typically, the root exudates contain acetate, succinate, lactate, fumarate, malate, citrate, isocitrate, aconitate, and oxalate. The release of these organic compounds increases microbial activity and nutrient exchange (Jones, 1998). Oxalotrophic bacteria metabolize oxalic acid, and the product of their metabolism leads to a strong local increase of soil pH (Martin et al., 2012). In *Arabidopsis thaliana* and *Phaseolus vulgaris* L., the degradation of oxalic acid has a protective function against pathogens, making the environment less favorable to fungi growth (Müller et al., 2016). Oxalate degrader microorganisms can increase the number of available phosphates influencing the phosphorus cycle and intensify the absorption of metals such as Fe and Al from soil (Morris and Allen, 1994). Other bacteria have been reported as oxalate degraders including *Burkholderia* spp., *Pseudomonas* spp., *Ralstonia*, and *Methylobacterium* spp. that we found as constituents of the core seed microbiome. Microbiome changes following leonardite treatment have already been studied in other plants, such as grapevine and potato (Cappelletti et al., 2016; Akimbekov et al., 2020). Also, Moreno et al. (2017) observed an increase of Gram-negative bacteria, such as *Proteobacteria*, as a consequence of the application of leonardite in barley.

The molecular analysis conducted in this work was done to evaluate hormonal gene responses, induced by leaf application of leonardite. The analyzed gene, belonging to hormonal metabolism, was selected among a larger set of 53 genes related to leonardite treatment on sugar beet and more generally based on the already known activity of humic acids on plant growth and development (Canellas et al., 2015; Nardi et al., 2016; Barone et al., 2019; Hajizadeh et al., 2019). However, the aforementioned genes were tested only on plants grown in hydroponic conditions, showing significant variation compared to untreated samples after 24 h of treatment. Thus, a first evaluation of the data obtained revealed the complexity of leonardite effects on sugar beet grown in a dynamic and variable context such as the open field. Among eight evaluated genes, the *LAX2* gene, encoding for auxin transport protein, showed a significant change between treated and untreated plants, while the others showed high variability among replicates. The overexpression of the *LAX2* transporter at 24 h from the foliar application could be explained as a particular consequence of the ascertained auxin-like activity of humic substances contained in the product (Pizzeghello et al., 2001; Canellas et al., 2002). However, 72 h from leonardite treatments, the increasing trend in *LAX2* expression of treated samples is no longer observable (data are not shown). High variability, due to the open-field growth conditions, was observed for the other hormone-related genes and, although they showed a high percentage of variation, the statistical test resulted in no significant difference. However, these auxin-like substances are mainly transported through the phloem but are also exported and imported from cell to cell thanks to specific membrane transporters (Petrášek and Friml, 2009). The movement of auxins and the regulation of homeostasis of these substances within the plants are key processes in the modulation of growth and development such as tropism, embryogenesis, and organogenesis of roots, shoots, and vascular tissues.

Regarding the relationship between sugar beet yield traits and leonardite treatment, we did not find significant differences in the impurity content between control and treated plants unlike Rahimi et al. (2020) who observed a decrease in Na, K, and α-amino N following treatment with humic acid. However, we reported higher values of sugar yield on treated plants. This improvement in production is confirmed also in other treated crops with higher tuber yield in potato, higher root growth and yield in tomato, and a higher dry matter in canola (Akinremi et al., 2000; Pertuit et al., 2001; Sanli et al., 2013).

The present study provides important evidence for understanding the effects induced by leonardite-based biostimulant in sugar beet. Initially, the microbial populations of plants grown under hydroponic and field conditions were compared. After leonardite treatment, the most responsive genus was *Oxalicibacterium*, comprising endophytes with plant growth-promoting activity. Also, an upregulation of the *LAX2* gene, coding for auxin transport proteins, has been observed. This finding is in agreement with our previous work (Barone et al., 2019), which was entirely conducted on hydroponics-grown seedlings and the same gene was overexpressed after leonardite treatment. A significant increase in sugar yield was also observed in plants treated with leonardite compared with untreated ones. Thus, the present study represents a step forward to understand the changes induced by leonardite-based biostimulant in sugar beet.

## Data Availability Statement

The datasets presented in this study can be found in online repositories. The name of the repository and accession numbers can be found below: European Nucleotide Archive (ENA) Browser, https://www.ebi.ac.uk/ena/browser/home, PRJEB42500 and ERP126366.

## Author Contributions

AB, FM, AS, and PS: conceptualization. GCo, LS, and FM: supervision. MCDL, GB, LM, GM, SR, MC, CB, and GCa: methodology. CB and PS: writing original draft. AR: writing, review, and editing. All authors contributed to the article and approved the submitted version.

## Conflict of Interest

FM was employed by company Sipcam Oxon S.p.A. GCa was employed by company COPROB. The authors declare that the research was conducted in the absence of any commercial or financial relationships that could be construed as a potential conflict of interest.

## Footnotes

1 http://bvseq.molgen.mpg.de
